# P-1316. Activity of Aztreonam-Avibactam and Comparators against Difficult-To-Treat Resistant (DTR) Enterobacterales from United States Medical Centers (2020-2024)

**DOI:** 10.1093/ofid/ofaf695.1504

**Published:** 2026-01-11

**Authors:** Helio SaderRodrigo E Mendes, Marisa Winkler, Dmitri Debabov, John Kimbrough, Mariana Castanheira

**Affiliations:** Element Iowa City (JMI Laboratories), North Liberty, IA; Element Materials Technology/Jones Microbiology Institute, North Liberty, Iowa; AbbVie, Chicago, Illinois; Element Iowa City (JMI Laboratories), North Liberty, IA; Element, North Liberty, IA

## Abstract

**Background:**

Aztreonam-avibactam (ATM-AVI) was recently approved by the United States (US) Food and Drug Administration (FDA) for the treatment of intra-abdominal infections. Difficult-to-treat resistant (DTR) isolates, defined as bacterial isolates expressing nonsusceptibility to all first-line agents, is a major problem worldwide. We evaluated the activity of ATM-AVI and comparators against DTR Enterobacterales from US medical centers.

**Table 1.**
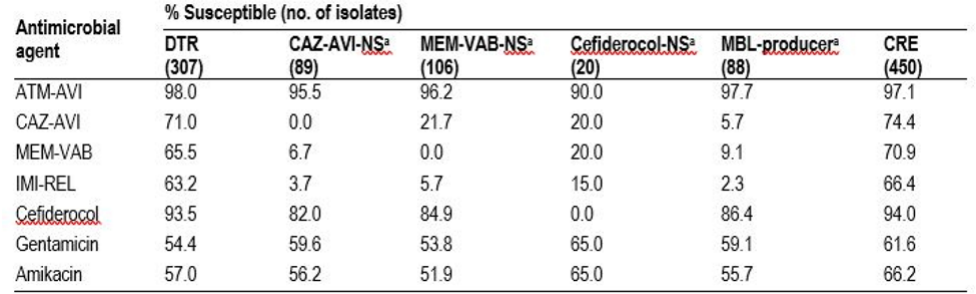
Antimicrobial susceptibility of selected resistant subsets a Includes only DTR isolates. Abbreviations: DTR, difficult-to-treat resistant; CAZ-AVI, ceftazidime-avibactam; NS, nonsuscpetible; MEM-VAB, meropenem-vaborbactam; CRE, carbapenem-resistant Enterobacterales.

**Figure:**
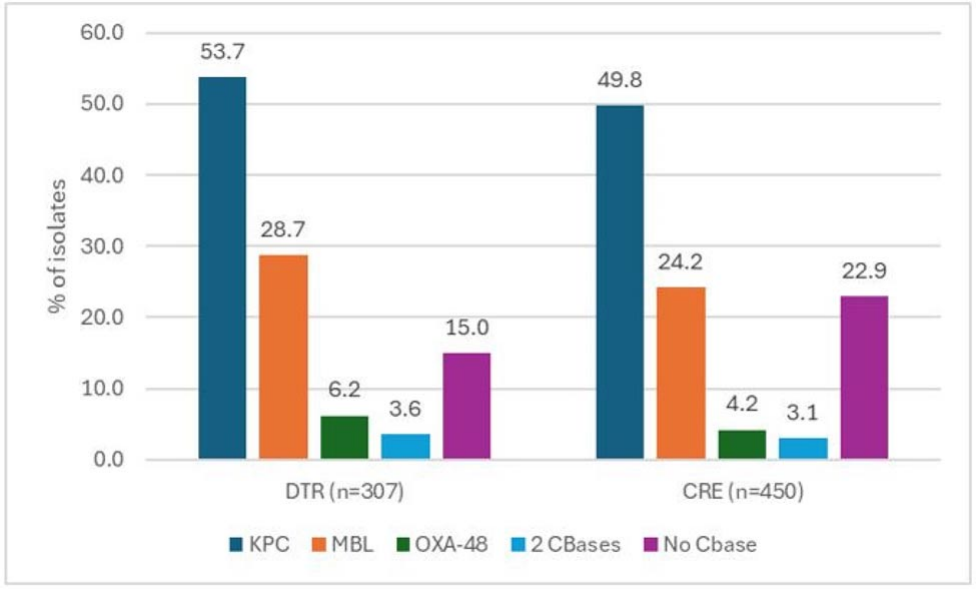
Distribution of carbapenemase (CBase) types among difficult-to-treat (DTR) and carbapenem-resistant (CRE) isolates

**Methods:**

42,295 Enterobacterales isolates were consecutively collected (1/patient) from 85 US medical centers in 2020-2024 and susceptibility tested by CLSI broth microdilution. The collection included 450 carbapenem-resistant (CRE; defined as resistant [R] to meropenem or imipenem) and 307 DTR (defined as a fluoroquinolone-R CRE) isolates; which were screened for β-lactamases by whole genome sequencing.

**Results:**

ATM-AVI was active (MIC ≤ 4 mg/L) against 98.0% of DTR (MIC_50/90_, 0.25/1 mg/L) and 97.1% of CRE (MIC_50/90_, 0.25/1 mg/L) isolates, and retained potent activity against DTR isolates nonsusceptible (NS) to ceftazidime-avibactam (CAZ-AVI; 95.5% susceptible [S]; MIC_50/90_, 0.25/1 mg/L), meropenem-vaborbactam (MEM-VAB; 96.2% S; MIC_50/90_, 0.25/1 mg/L), and/or cefiderocol (90.0% S; MIC_50/90_, 0.5/4 mg/L; Table 1). Cefiderocol was active against 93.5% of DTR isolates, whereas CAZ-AV, MEM-VAB, IMI-REL and the aminoglycosides exhibited limited activity against these organisms. ATM-AVI (MIC_50/90_, 0.12/0.5 mg/L and cefiderocol (MIC_50/90_, 2/8 mg/L) were active against 97.7% and 86.4% of MBL producers, respectively. The most common carbapenemase (CBase) gene identified among DTR isolates were *bla*_KPC_ (53.1% of isolates) and *bla*_NDM_ (25.7%). DTR and CRE isolates exhibited similar frequencies of CBase types. An MBL gene was observed in 27.4% of DTR and 24.2% of CRE isolates (Figure 1).

**Conclusion:**

ATM-AVI retained potent activity against DTR Enterobacterales from US medical centers and its activity was not adversely affected by clinically relevant CBases or resistance to agents used to treat multidrug-resistant Enterobacterales. The activities of other β-lactamase inhibitor combinations and cefiderocol were compromised by the increased occurrence of MBL producers among DTR and CRE isolates.

**Disclosures:**

Helio Sader, United States Food and Drug Administration: FDA Contract Number: 75F40123C00140 Rodrigo E. Mendes, PhD, GSK: Grant/Research Support|Shionogi & Co., Ltd.: Grant/Research Support|United States Food and Drug Administration: FDA Contract Number: 75F40123C00140 Marisa Winkler, MD, PhD, Basilea: Advisor/Consultant|Basilea: Grant/Research Support|GSK: Advisor/Consultant|GSK: Grant/Research Support|Melinta Therapeutics: Advisor/Consultant|Melinta Therapeutics: Grant/Research Support|Mundipharma: Advisor/Consultant|Mundipharma: Grant/Research Support|Pfizer: Advisor/Consultant|Pfizer: Grant/Research Support|Pulmocide: Advisor/Consultant|Pulmocide: Grant/Research Support Mariana Castanheira, PhD, Melinta Therapeutics: Advisor/Consultant|Melinta Therapeutics: Grant/Research Support

